# Staining with methylthioninium chloride for the diagnosis of fungal keratitis

**DOI:** 10.3892/etm.2013.1288

**Published:** 2013-09-05

**Authors:** LAN LAN, FENG-YUN WANG, GUANGWEI ZENG

**Affiliations:** 1 Department of Ophthalmology, First Affiliated Hospital of Henan University of Science and Technology, Luoyang, Henan 471003, P.R. China; 2 Department of Plastic, Reconstructive and Burns Surgery, First Affiliated Hospital of Henan University of Science and Technology, Luoyang, Henan 471003, P.R. China

**Keywords:** fungal keratitis, methylthioninium chloride staining, KOH-based smear, culture

## Abstract

The aim of this study was to investigate the potential efficacy of staining with methylthioninium chloride (MC) for the diagnosis of fungal keratitis. A total of 70 cases of fungal keratitis were included in the study from January 2009 to December 2010. The corneal scraping specimens of the patients were collected and stained with MC or a 10% potassium hydroxide (KOH)-based smear prior to microscopic examination. The staining results were confirmed with fungal culture and strain identification, which are recognized as ‘gold standards’ for the diagnosis of fungal keratitis. Among the 70 cases of fungal keratitis, 58 cases were positive and the positive rate was 82.86%. MC staining showed a positive rate of 62.86%, with higher levels of sensitivity (70.69%) and specificity (34.61%) compared with staining with the 10% KOH-based smear. The KOH-based smear showed a positive rate of 44.29% (P<0.05), a level of sensitivity of 44.83% (P<0.05) and a specificity of 17.95% (P<0.05). In addition, the MC staining showed false positive and negative rates of 25.00 and 29.31%, respectively, an accuracy index of 5.30% and positive and negative predictive values of 93.18 and 34.61%, respectively. The results indicate that MC staining is a fast and effective method for the early diagnosis of fungal keratitis.

## Introduction

Fungal keratitis is a serious ocular disease leading to blindness that frequently occurs among the agricultural population of developing countries, such as China and India. In recent years, the incidence of the disease has tended to increase, and it has become the most common infectious corneal disorder in certain regions (1–5). Earlier and accurate diagnosis of the fungal infection in the cornea is crucial for effective treatment and the prevention of blindness. However, the diagnosis of fungal keratitis remains dependent upon the laboratory examination. At present, the diagnosis is dominated by smear cytology and fungal cultures (6–8). The ‘gold standards’ for the diagnosis of fungal keratitis are fungal culture and strain identification (5); however, these are lengthy processes, usually taking 2–21 days, and making a correct diagnosis often delays clinical treatment (9–11). The traditional potassium hydroxide (KOH)-based smear shows a low positive rate in the diagnosis of fungal keratitis, and thus a high rate of misdiagnosis. The rapid and accurate diagnosis of fungal keratitis remains a focus of current investigations (8). To the best of our knowledge, there have been no previous studies regarding the use of a methylthioninium chloride (MC) stain for the diagnosis of corneal fungal keratitis. We suggested the concept based on the ability of MC to detect gonococcus, thereby enabling the diagnosis and treatment of gonorrhea (12), and the fact that an injection of MC in early-stage breast cancer enables the timely detection of metastasized cancer cells in sentinel lymph nodes. With regard to its use in cancer, the MC stain is sprayed directly under the endoscope to detect early cancer cells, in addition to diagnosing pre-cancerous pathological changes and early gastric cancer. However, MC has not been used for the detection of keratitis infected by fungi. The aim of the current study was to investigate the efficacy of MC staining for the diagnosis of fungal keratitis. A total of 70 cases of fungal keratitis were analyzed by MC staining and the positive rates, sensitivity and specificity were compared with those of a 10% KOH-based smear.

## Materials and methods

### Patients

Seventy patients diagnosed clinically with fungal keratitis were admitted to The First Affiliated Hospital of Henan University of Science and Technology (Luoyang, China) between January 2009 and December 2010. The patients included 54 males and 16 females, with an average age of 52.2 years (range, 5–83 years). The involved eye was OD (right) in 38 cases and OS (left) in 32 cases. The occupation of the patients was ‘peasant’ in 47 cases (67.1%), ‘worker’ in 14 cases (20%) and ‘other’ in nine cases (12.9%). The majority of the patients (53 cases, 75.7%) had a history of ocular trauma; among these 53 cases, 35 (66%) cases were injured by plants and other such foreign bodies, such as grains, grass, pieces of wood, bamboo and leaves. In addition, 11 (15.7%) cases had soil contamination, three cases were injured by welding sparks and four cases were due to other causes. Seven patients had a medical history of systemic administration of glucocorticoids and immunosuppressive agents for systemic disorders. This study was conducted in accordance with the Declaration of Helsinki and with approval from the Ethics Committee of the Henan University of Science and Technology. Written informed consent was obtained from all participants.

### Sample collection

The affected eyes were treated with the topical anesthetic Benoxil (Invitrogen Life Technologies Carlsbad, CA, USA), and the skin around the eye was sterilized with 75% alcohol. The eye was opened with eyelid retractors. The surface, peripheral parts and basal substance of the corneal ulcer were scraped with a sterilized lancet and collected as smears for MC staining (Invitrogen) and KOH-based smears, in order to detect the fungus. The specimens were also sent for culture of the fungus.

### Detection of the fungus by KOH-based smears

The specimens scraped from the corneal ulcer were removed and spread on slides to form as thin a layer as possible. One drop of 10% KOH was added to each slide, and a coverglass was used to cover the slide for 10 min in order to remove impurities. Following this, a low-power microscope was initially used to locate the species position, prior to a high-power microscope being used to detect the hyphae and spores.

### Detection of the fungus by MC staining

The specimens scraped from the corneal ulcer were removed and spread on slides. MC (20 *μ*l) was added to each slide and a coverglass was used to cover the slide for 5 min for staining. Following this, a low-power microscope was initially used to locate the species position, prior to a high-power microscope later being used to detect the hyphae and spores.

### Fungal cultivation

An appropriate quantity of ulcer extract was collected and then inoculated in a tube of Sabouraud dextrose agar (Gibco Co., Gaithersburg, MD, USA) at 25°C for 3–21 days. The cultures were observed at certain intervals for fungal growth. The strains of fungi were identified according to the appearance of the colony, growth rate and the morphological characteristics of the hyphae, spores or bacteria.

### Data collection and statistical analysis

The result of the fungal cultivation was used as the ‘gold standard’ for the diagnosis of fungal keratitis. The results from the MC staining and KOH-based smears were analyzed to determine the positive rate, sensitivity, specificity, false positive rate, false negative rate, accuracy index, positive predictive value and negative predictive value for the fungi.

Analyses were performed using SPSS for Windows version 10.0 (SPSS, Inc., Chicago, IL, USA), with tests including the Wilcoxon non-parametric rank sum test and the Spearman rank related test. P<0.05 was considered to indicate a statistically significant difference.

## Results

**Figure 1.**
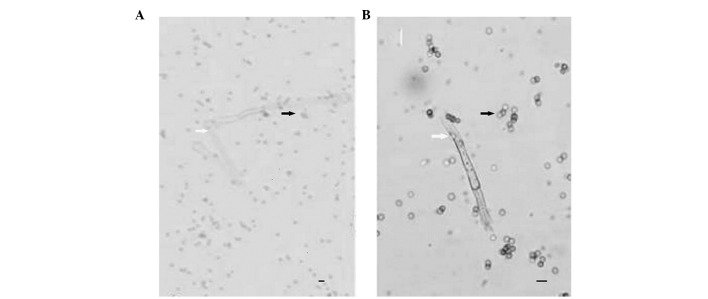
Fungus from specimens of corneal ulcer, detected by smears. (A) Potassium hydroxide (KOH)-based smears (magnification, ×100); (B) methylthioninium chloride (MC) staining (magnification, ×200). Bar, 5 *μ*m; white and black arrows indicate hyphae and spores, respectively.

The fungal hyphae in the KOH-based smears were visible as transparent streak-structures, which were poorly contrasted against the background (Fig. 1A). The fungal hyphae observed using MC staining were blue tubular structures, which were strongly contrasted against the background (Fig. 1B). Among the 70 cases clinically diagnosed with fungal keratitis, 58 cases were shown by fungal culture and strain identification to have fungi, and the positive rate was 82.86%. The positive rate obtained with the MC staining was 62.86%, with a sensitivity of 70.69% and a specificity of 34.61%. The false positive and negative rates were 25.00 and 29.31%, respectively, the accuracy index was 5.30% and the positive and negative predictive values were 93.18 and 34.61%, respectively (Table I). The positive rate obtained with the KOH-based smears was 44.29%, the sensitivity was 44.83% and the specificity was 17.95% (P<0.05). The false positive and negative rates were 41.67 and 55.17%, respectively, the accuracy index was −37.22% and the positive and negative predictive values were 83.87 and 17.95%, respectively (Table II).

## Discussion

Fungal keratitis is a serious blindness-causing keratopathy that occurs as a result of a disease-causing fungus. It is difficult to diagnose in the clinic and is easily misdiagnosed, resulting in blindness due to improper treatment. Under normal circumstances, the fungus does not invade the healthy cornea. However, under certain conditions, such as when ocular trauma occurs or surgery is performed, non-disease-causing fungi may become pathological, giving rise to a corneal secondary fungal infection. Pathological infection may also occur when antibiotics, corticosteroids or immunosuppressants are administered over a long period, when the resistance of the body is reduced or when keratitis or dry-eye symptoms occur. It may also arise due to the cornea becoming infected by fungi or fungal crops, such as cereals and dry grasses, or as a result of wheat stubble injuries. The most common type of disease-causing organism is *Aspergillus*, followed by *Fusarium* toxin, *Candida albicans*, *Bacillus* and *Streptothrix* (13,14). Due to the fact that disease-causing strains are different, the corneal ulcer morphology varies.

In recent years, the incidence of fungal keratitis has increased annually and the infection has become an increasingly serious problem. Therefore, early diagnosis and treatment have a decisive effect on the prognosis of the disease, particularly with regard to eyesight. A preliminary diagnosis may be made according to a history of infection following corneal injury by plants, and in combination with the characteristics of corneal focus. However, the accurate diagnosis of fungal keratitis continues to depend upon the laboratory examination (8). Gram and Giemsa staining of corneal scraping specimens, and hematoxylin and eosin staining are commonly used methods at the early stage of diagnosis, but these stainings lack sufficient efficacy. The fungal culture may involve the administration of a blood agar base or a chocolate medium. If corneal smears and fungal cultures are all negative, despite fungal keratitis being highly suspected in the clinic, a corneal tissue biopsy may be taken into account; however, these methods are all lengthy procedures, which delay the diagnosis. In addition to immunofluorescence staining, confocal laser microscopy and polymerase chain reaction (PCR) technology represent a better prospect for application in the field of fungal keratitis diagnosis, although these methods are expensive, which reduces their popularity and use (15–17). When concerned with medical disputes and legal disputes, it is usually possible to reach an accurate conclusion through pathological diagnosis, and, therefore, pathological diagnosis represents the final verdict of diagnosis (17). The early, rapid and effective diagnosis of fungal keratitis is a focus of current studies (1,8).

MC is widely used in clinics as a chemical indicator, a dye, a biological staining agent and a biological antidote. MC is used to stain various organs and has such advantages as few side-effects, a low cost, a large range of doses and a high accuracy rate. Therefore, it is suitable for wider popularization. MC has numerous differences from other dyes, including its ability to stain finer lymphatic vessels, its clear blue staining of sentinel lymph nodes (SLN), its relatively low molecular weight and its rapid excretion rate. Therefore, it is used more skillfully, and rapidly (18). It has been demonstrated that an overdose of MC has certain harmful effects on tissues and brings about tissue necrosis. However, in the present study, MC was promptly removed following its use for staining and, therefore, there were no side-effects. Thus, MC was used successfully as a dye in this group of cases. MC is a blue liquid, which colors hyphae and spores purple, thereby enhancing their contrast against the background and enabling them to be detected easily. This improves the sensitivity, positive rate and the positive predictive value of the diagnostic experiment. Hyphae stained with MC may be easily distinguished from corneal fibers and impurities.

In the present study, we demonstrated that the sensitivity, specificity, positive rate, accuracy index and positive and negative predictive values of the fungi detected by MC staining were higher than those obtained using KOH-based smears. The false positive and negative rates for the fungi obtained using MC staining were lower than those obtained using KOH-based smears. The authenticity of the diagnostic experiment includes its sensitivity and specificity. The sensitivity, i.e., true positive rate, means that the diagnostic experiment accurately identifies the true non-keratitis cases when the disease is not present. The higher the sensitivity and specificity, the higher the accuracy index and the authenticity of the diagnostic experiment. It was demonstrated in this study that the sensitivity, specificity and accuracy index of the detection of fungi by MC staining were higher than those by KOH-based smears, suggesting that the MC staining had a higher authenticity in the detection of fungi. The positive predictive value refers to the proportion of true positive results among the positive cases in the diagnostic experiment, while the negative predictive value refers to the proportion of true negative results among the negative cases in the experiment. Investigations into the level of disease rate in the population have a great impact on the positive predictive value of a diagnostic experiment. The population in the present study included patients clinically diagnosed with fungal keratitis, i.e., belonging to a high-risk population, and, therefore, the positive predictive value of the diagnostic experiment was relatively high. The MC staining was demonstrated to have a higher positive predictive value than that of the KOH-based smears, reaching 93.18%. This indicated that the MC staining had a greater chance of detecting the individuals positive for fungi among the cases with fungal keratitis, enabling the guidance of early clinical treatment. The false positive rate is the rate at which the diagnostic experiment wrongly judges the true non-keratitis cases to be cases with the disease, i.e., the misdiagnosis rate. The false negative rate is the rate at which the diagnostic experiment wrongly judges the true keratitis cases to be cases without the disease, i.e., the rate of missed diagnosis. It was demonstrated in this study that the false positive and negative rates for fungi were lower with MC staining than with KOH-based smears, indicating that the rate of misdiagnosis and the rate of missed diagnosis by MC staining were lower, respectively.

Several factors affecting the detectable rate of fungal smears were considered, one of which was species collection. When fewer fungi exist in the smears, the detectable rate by MC staining decreases (5). Fungal growth is most often located in the peripheral ulcer sites and the bottom parts of the ulcer, and the sticky white substance in the center of the ulcer is often the hyphal plexus. There are relatively few corneal species. Therefore, the sample containing the white sticky substance in center of ulcer was collected, in order to improve the detection rate of fungal smears. The other factor was over-thickness of the smears. When the smears are over-thick, overlapping of the species causes microscopic evaluation to become more challenging; therefore, the smear species should be dispersed as much as possible in the staining liquid, and if necessary, pressure should be applied to the glass coverslip, in order to disperse the mass-like substances.

The prognosis of fungal keratitis, with regard to eyesight, is poor. Only in cases where there is a small corneal focus that is diagnosed in a timely manner and effectively treated is the restoration of sight certain. An increase in the severity of the condition may endanger the eyeball and eventually lead to blindness (8). The results of the present study showed that the outcome of cultivation was the ‘gold standard’ for confirming the diagnosis of fungal keratitis. Among the 70 cases with fungal keratitis, 58 cases were observed to possess fungi, and the positive rate was 82.86%. The positive rate obtained with MC staining was 62.86%, which indicated that the MC staining had a lower positive rate than cultivation. However, MC staining, with its low cost, rapid detection of fungi (within a few minutes), high sensitivity and higher relative positive predictable value, remains a simple, rapid and effective technique for the early diagnosis of fungal keratitis. The present study has conducted preliminarily investigations of the technique using morphological characterization; however, further investigations involving molecular biology are required.

## Figures and Tables

**Table I.** Comparison between fungal detective rates of fungal culture and methylthioninium chloride (MC) staining.

MC staining	Fungal culture		Total
	Positive	Negative	
Positive	41	3	44
Negative	17	9	26
Total	58	12	70

**Table II.** Comparison between fungal detective rates of fungal culture and potassium hydroxide (KOH) staining.

KOH-based smears	Fungal culture		Total
	Positive	Negative	
Positive	26	5	31
Negative	32	7	39
Total	58	12	70
